# The Matrix Reloaded: The Hepatic Matrisome as a Therapeutic Opportunity to Fight Liver Fibrosis

**DOI:** 10.3390/biom16060884

**Published:** 2026-06-16

**Authors:** Cristina Benavides, Pepa Kecheva, Fernando Solano, Olga Martínez-Arroyo, Juan V. Esplugues, Ana Blas-García, Nadezda Apostolova

**Affiliations:** 1 Departamento de Farmacología, Facultad de Medicina, Universitat de València, 46010 Valencia, Spain; crisbetu@uv.es (C.B.); fersogui@gmail.com (F.S.); olga.martinez-arroyo@ext.uv.es (O.M.-A.); juan.v.esplugues@uv.es (J.V.E.); 2 FISABIO–Hospital Universitario Dr. Peset, 46017 Valencia, Spain; ana.blas@uv.es; 3 Departamento de Fisiología, Facultad de Medicina, Universitat de València, 46010 Valencia, Spain; kepe@uv.es; 4 CIBERehd (Centro de Investigación Biomédica en Red de Enfermedades Hepáticas y Digestivas), Instituto de Salud Carlos III, 46010 Valencia, Spain

**Keywords:** liver fibrosis, hepatic stellate cells, therapeutic targets, extracellular matrix, collagen

## Abstract

Liver fibrosis is the excessive accumulation of extracellular matrix (ECM) that occurs in most types of chronic liver diseases (CLDs) as a response to sustained liver injury. While the ECM comprises different proteins, collagen being the most abundant, the term matrisome refers to a plethora of ECM-related molecules, including collagen-associated proteins, growth factors, cytokines, enzymes and their endogenous inhibitors. The hepatic matrisome undergoes significant qualitative and quantitative changes during liver fibrosis. Despite intense research over recent years, our understanding of the matrisome in the liver—both in health and disease—and particularly of its function beyond its conventional structural role, remains poor. This review highlights how comprehending hepatic matrisome responses to liver injury can yield novel insights into disease progression and regression and could be exploited as a potential antifibrotic strategy. The antifibrotic potency of drugs that interfere with the matrisome at different levels has been demonstrated in preclinical studies, but translation to clinical trials remains still limited. So far, simtuzumab (LOXL2 inhibitor antibody), imatinib (small-molecule inhibitor against discoidin domain receptors—DDRs), bexotegrast (integrin inhibitor), GR-MD-02 (galectin 3 inhibitor), and BMS-986263 (siRNA-targeting HSP47) have been or are being evaluated in clinical trials related to CLD, and some of them have shown promising results.

## 1. Introduction

Chronic liver disease (CLD) is a term that englobes a spectrum of pathologies associated with prolonged liver injury (classified as hepatotoxic or cholestatic injury) and which are characterized by progressive liver tissue damage and chronic inflammation [1,2]. The primary causes of CLD include viral hepatitis, alcoholism, obesity/metabolic syndrome, autoimmune conditions, and genetically determined metabolic abnormalities. Liver fibrosis (LF), a common feature of nearly all forms of CLD, is an aberrant, albeit reversible (to a certain degree), wound-healing response characterized by the excessive accumulation of extracellular matrix (ECM) [3], which leads to architectural distortion and the replacement of functional hepatic parenchyma with non-functional scar tissue. This pathological process typically progresses and can ultimately lead to cirrhosis, a severe stage of CLD defined by the formation of regenerative nodules and distortion of the hepatic vasculature and portal hypertension. These structural changes and chronically impaired liver function further increase the risk of hepatocellular carcinoma (HCC), the most frequent form of primary liver cancer [4]. Thus, cirrhosis is highly prevalent among patients with HCC, regardless of the underlying type of liver disease [5]. Over the past few decades, CLD has emerged as one of the most prevalent pathologies [6,7] and one of the major causes of death worldwide [8]. Global statistics estimate that it accounts for approximately 2 million deaths annually (4% of global mortality) [9], with cirrhosis and liver cancer causing 1.16 and 0.79 million deaths, respectively, representing the 11th and 16th most common causes of death each year [10].

Even though research on hepatic antifibrotic drugs is a ‘hot topic’, there is to date no specific clinically approved drug for the treatment of LF. Currently available medicines are used to treat the underlying causes of liver injury and primary diseases, such as antiviral drugs in the case of viral hepatitis or lipid-lowering and antidiabetic drugs for metabolic dysfunction-associated steatotic liver disease (MASLD), previously known as non-alcoholic fatty liver disease (NAFLD). Importantly, these treatments have proven that LF is a reversible process, which is particularly patent in the case of antiviral therapy for hepatitis infection. For example, in one study with a cohort of patients with chronic hepatitis C virus (HCV) infection in whom a virological response was sustained for at least five years, direct acting antiviral (DAA) therapy was highly effective in achieving LF regression and decreasing mean FibroScan^®^ values [11]. However, patients with stage 4 fibrosis (F4) experience less LF regression [11] and severe stages of CLD are very difficult to manage; indeed, the only treatment for a failing liver is a liver transplant. Given this lack of specific therapies, recent years have seen a remarkable amount of preclinical and clinical research focused on the pursuit of novel therapeutic options and the repositioning of already existing drugs. At present, the main strategies being explored to combat CLD include control of inflammation and the immune response, interference with the activation of hepatic stellate cells (HSCs) and their deactivation/clearance, and improvement of the cell injury in liver parenchyma, including the regulation of apoptosis. Interference with ECM synthesis and degradation has also emerged as a promising target. The present review aims to explore the relevance of the ECM/matrisome in LF and the processes and mechanisms that might form part of the development of antifibrotic drugs.

## 2. Liver Fibrogenesis: The Role of Hepatic Stellate Cells and Beyond

**Figure 1:**
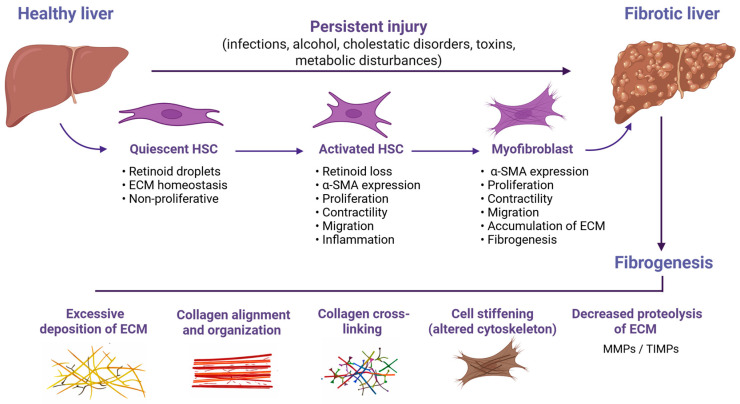
Pathogenesis of liver fibrosis. Liver fibrosis arises from a wide range of etiologies and results from an imbalance between fibrogenesis (production of extracellular matrix) and fibrolysis (its degradation). Activation of hepatic stellate cells and their transformation into myofibroblasts represent the central pathogenic mechanism driving this process. α-SMA: alpha smooth muscle actin; ECM: extracellular matrix; HSC: hepatic stellate cell; MMPs: matrix metalloproteinases; TIMPs: tissue inhibitors of matrix metalloproteinases. Created in BioRender. Benavides, C. (2026) https://BioRender.com/yxw1ykr.

Active myofibroblasts are the main drivers of the process of fibrogenesis due to their ability to increase the expression, secretion and deposition of ECM components upon sustained liver damage [12,13]. Of note, HSCs, which are located in the space of Disse and account for 5–8% of the total number of hepatic cells [14], are the predominant source of ECM-producing myofibroblasts [15]. Other cells, such as bone marrow-derived fibrocytes and portal vein fibroblasts, also generate the matrix in LF, albeit to a lesser degree [16,17]. During the process of activation, HSCs undergo transformation from quiescent, vitamin A-rich HSCs (qHSCs) into active HSCs (aHSCs), which are proliferative, migratory, contractile and devoid of vitamin A droplets (Figure 1). Besides these two classic phenotypes, several studies have highlighted major HSC heterogeneity [18]. For instance, single-cell RNA sequencing in livers from mice with non-alcoholic steatohepatitis (NASH) revealed the presence of four distinct HSC clusters: the classic fibrogenic myofibroblast, a proliferating cluster, an intermediate activated cluster, and an immune/inflammatory cluster [19].

aHSCs release pro-inflammatory, pro-fibrogenic, and pro-mitogenic cytokines, thus interacting with other cells involved in liver inflammation [20]. The process of HSC activation occurs in 2 phases, initiation and perpetuation, and involves a complex network of numerous mitogenic, fibrogenic and inflammatory pathways, including oxidant stress signals and soluble autocrine and paracrine fibrogenic factors emanating from HSCs and adjacent cells [20], among which transforming growth factor-beta (TGF-β) and platelet-derived growth factor (PDGF) are considered to be pivotal [13,21].

Several transcription factors help maintain the quiescent phenotype of HSCs, such as peroxisome proliferator-activated receptor (PPAR)γ, retinoic acid receptors (RARs), retinoid X receptors (RXRs), the pregnane X receptor (PXR), and LIM/homeobox 2 protein (LHX2), while others, including Kruppel-like factor 6 (KLF6), Gα-interacting, vesicle-associated protein/Girdin (GIV/Girdin), and methyl-CpG-binding protein 2 (MeCP2), drive the pro-fibrotic transformation of qHSCs. Given the crucial role of aHSCs in LF, reducing their number is essential for reversing and treating LF. The elimination of aHSCs occurs through three major pathways: a return to a quiescent phenotype; cell death (mainly apoptosis); and senescence [22,23,24].

The development of LF is a complex process in which many cell types (parenchymal and non-parenchymal) participate [25]. Under basal conditions, almost all hepatocellular cells (hepatocytes, cholangiocytes and sinusoidal endothelial cells) contribute to the ECM, while activated myofibroblasts are the primary producers of ECM components in the injured liver. aHSCs are considered the major source of ECM in LF; nevertheless, in early, cholestatic, or specific injury models, significant contributions are made by other cell types, such as portal fibroblasts, bone marrow-derived cells, hepatocytes, and immune cells. Besides HSCs, liver sinusoids also contain liver sinusoidal endothelial cells (LSECs), a fenestrated cell type without an organized basement membrane, and immune cells, such as Kupffer cells (KCs) and hepatic natural killer cells. HSCs communicate with these cell types via bone morphogenetic protein (BMP)- 9 and BMP-10 to maintain their identity and function, thereby influencing liver zonation and iron metabolism. The opposite regulation is also relevant, as LSECs in the healthy liver maintain HSCs in a quiescent state via vascular endothelial growth factor (VEGF)-stimulated NO production. During liver injury, LSECs lose their fenestration, form continuous basal membrane, and develop inflammatory and fibrotic features. Such capillarized LSECs are on the one hand permissive to HSC activation and on the other hand are an active contributor to the process of fibrogenesis through synthesis of collagen and fibronectin [26]. KCs are key to the process of LF and play a dual role: while they participate in the activation of qHSCs upon liver injury through the release of pro-inflammatory cytokines such as TNF-α, IL-1β, and TGF-β [18], they also contribute to LF resolution through the production of a large spectrum of matrix metalloproteinases (MMPs) [27].

## 3. The Matrisome: Another “Some”

The ECM is a dynamic and complex 3D network of more than 300 different interlinked proteins and other polymers (polysaccharides) outside of cells, and it plays a crucial role during organ development, tissue repair, multiple physiological and pathophysiological processes, and aging. It accounts for over 20% of human bodyweight and plays a pivotal role in maintaining tissue architecture and homeostasis, providing essential mechanical support to organs and enabling communication between the surrounding cells and their microenvironment [28]. Besides this structural role, ECM is key to multiple pathways of cellular signaling, as it interacts with cells through cell adhesion receptors, thereby influencing cellular processes like survival, proliferation, migration and differentiation, in response to internal and external stimuli. Among the cellular receptors that bind to the ECM are many proteins: integrins, discoidin domain receptors (DDRs), syndecans, CD44, the receptor for hyaluronic acid-mediated motility, the leukocyte receptor complex (leukocyte-associated immunoglobulin-like receptor 1, LAIR-1), urokinase plasminogen activator receptor-associated protein (uPARAP) and Toll-like receptor 4 (TLR4). The ECM can be subdivided into two main categories: the interstitial matrix and the basement membrane, which play a variety of roles depending on the organ and tissue location. Dysregulation of ECM components has been implicated in a range of pathological conditions, including cancer, genetic disorders, abnormal stem cell behavior, atherosclerosis and fibrosis [29,30]. This is why ECM constitutes a potential diagnostic tool and biomarker for various diseases; for example, the evaluation of ECM components can help diagnose and monitor fibrosis and cardiovascular diseases [31,32], while the modulation of ECM composition represents a potential therapeutic target [33,34].

The main components of ECM are various fibrous proteins, such as collagens and elastin; adhesive glycoproteins, such as extracellular matrix protein 1 (ECM1), fibronectin, vitronectin, tenascin C and laminins; proteoglycans (e.g., decorin, perlecan, versican), which consist of core proteins linked to glycosaminoglycans (e.g., chondroitin sulfate, dermatan sulfate, heparan sulfate, keratan sulfate); and free glycosaminoglycans, such as hyaluronan. ECM proteins comprise independently folded regions characterized by highly conserved sequences and structures. These domains interact with adhesion receptors such as integrins to transmit signals to cells and facilitate cell–matrix attachment [35]. In addition to ECM constituents, there are collagen-associated proteins, ECM-regulating and modifying enzymes (e.g., lysyl oxidases and proteases), and ECM-resident storage molecules, such as cytokines, growth factors and morphogens (e.g., PDGF, VEGF and TGF-β), for which the ECM acts as a reservoir. Actually TGF-β1 is secreted and stored by ECM molecules to which it covalently attaches, forming an inactive protein complex known as latent TGF-β1 (LTGF-β1). Collagen-associated proteins include galectins, annexin A2 (ANXA2) and secreted protein acidic and rich in cysteine (SPARC). Although the roles of these proteins in the liver are not fully understood, they are known to act as molecular modulators of cell–matrix adhesion, immune-cell recruitment and angiogenic remodeling. All the mentioned components collectively constitute what is referred to as the matrisome. Thus, the matrisome can be divided into the core matrisome (the actual ECM) and the associated matrisome, which includes proteins that enable ECM remodeling or associate with ECM, such as ECM receptors [36]. Indeed, the number of proteins that are known to belong to the matrisome is growing; through proteomic analyses of in vivo ECM composition and in silico prediction, 278 genes representing 1% of the human proteome [37] have been discovered to be essential components of the human matrisome.

The matrisome is fundamental for multiple functions and processes, such as cell signaling, and tissue regeneration and repair. ECM offers a scaffold that directs cell migration and encourages tissue rebuilding following tissue injury. During tissue repair, the matrisome undergoes dynamic changes related to ECM remodeling and consisting of the breakdown of old matrix components and the synthesis of new ones. Numerous bioactive and signaling compounds can be sequestered within the ECM and are subsequently released in a regulated manner to modulate cellular function. Additionally, intracellular signaling cascades can be influenced by the physical properties of the ECM, such as stiffness and rigidity.

In the liver, the matrisome plays a pivotal role in regulating key physiological processes essential for homeostasis, such as blood flow, nutrient exchange, and waste elimination. In the context of hepatic injury, it orchestrates effective tissue repair and regeneration and is also crucial for fibrogenic remodeling. Increased density and stiffness of the liver and higher resistance to fibrolysis are all effects of excessive fibrogenesis, ECM deposition and cross-linking [38]. Interestingly, changes in the matrisome occur before major alterations in liver tissue. It exhibits dynamic responses to acute lipopolysaccharide (LPS) exposure and chronic ethanol-induced stress well before the appearance of overt fibrotic changes in the liver. The pathological responses to these stressors may, in part, be driven by alterations in the matrisome itself [39]. Actually, though there is abundant evidence of numerous matrisome components in association with the fibrotic tissue in LF, their exact implication in LF is unclear. In this regard, different components of the matrisome exert pro-resolutive or pro-fibrotic effects. For example, ECM1 attenuates LF, as it inhibits LTGF-β1 activation mediated by TSP-1, ADAMTS1 (A disintegrin and metalloproteinase with thrombospondin motifs 1), MMP-2 and MMP-9 [40] and binds to CTGF, thus blocking integrin αvβ6-mediated TGF-β activation [41]. Syndecan-1, a proteoglycan, inhibits the early stages of liver fibrogenesis by interfering with TGF-β1 action and upregulating *MMP14* [42]. Similarly, microfibrillar-associated protein 2 (MFAP-2), a component of the ECM, prevents LF in mice by regulating ECM stabilization, focal adhesion signaling and inflammation [43]. Of note, while *Mfap2* ablation was shown to have minimal impact on collagen deposition during CCl_4_ exposure, it significantly delayed LF regression after CCl_4_ cessation. Conversely, CREBZF (CREB/ATF bZIP transcription factor related to hepatic steatosis and metabolic defects) induces HSC activation and LF in a hepatocyte-autonomous manner by stimulating the ECM protein osteopontin, thereby operating as a checkpoint for steatosis-to-NASH progression [44].

The ECM is continuously being remodeled, and this includes a strictly regulated process of ECM degradation. Due to the tightly packed helical structure of fibrillar collagens most proteolytic enzymes are unable to degrade them. Under physiological conditions, intracellular degradation of fibrillar collagen is pivotal in maintaining collagen homeostasis, and occurs via internalization, phagocytosis, micropinocytosis, or uPARAP-mediated endocytosis. Extracellular degradation of ECM, which predominates under pathological conditions, is mediated by different enzymes, especially proteases, among which MMPs are crucial. Their actions are controlled by tissue inhibitors of metalloproteinases (TIMPs).

MMPs are a family of more than 24 zinc-dependent membrane-bound and secreted endopeptidases that can degrade any component of the ECM [45]. According to their substrate specificity, MMPs have been divided into five categories, collagenases (MMP-1, MMP-8, MMP-13, MMP-18), gelatinases (MMP-2, MMP-9), matrilysins (MMP-7, MMP-26), stromelysins (MMP-3, MMP-10) and membrane-type (MMP-14, MMP-15, MMP-16, MMP-17, MMP-24, MMP-25), while the remaining 6 MMPs belong to the unclassified group of “other MMPs”. Importantly, a range of ECM-derived fragments known as matrikines—and generated under different proteolytic conditions—mediate multiple signaling functions, including tissue remodeling and fibrogenesis [46,47]. An example of fibrosis-related matrikine is endostatin, a hydrolysis product of collagen type XVII with anti-angiogenic properties that downregulates multiple pro-fibrotic pathways, including TGF-β1 signaling in the liver [48]. MMPs not only directly degrade ECM components, but also act on non-ECM substrates, such as chemokines and cytokines, thus modulating the cell inflammation and signaling pathways involved in fibrogenesis [49]. For instance, they cleave LTGF-β1 complexes, releasing active TGF-β. Active TGF-β stimulates HSC activation, ECM synthesis, and the production of TIMPs, creating a positive feedback loop that sustains fibrogenic responses in the liver. Similarly, hepatocyte growth factor (HGF), a key promotor in the regeneration of hepatocytes following injuries, is bound with heparin-like proteoglycans in its inactive form [50]. MMPs, particularly MMP-2, MMP-9, and MMP-13, are upregulated in response to inflammatory and pro-fibrotic signals, promoting ECM degradation and turnover [45]. Dysregulated MMP-mediated ECM degradation disrupts the balance between ECM synthesis and degradation, leading to progressive LF.

TIMP is a family of at least four physiological inhibitors (TIMPs 1–4) that operate as endogenous suppressors of MMPs, thus regulating excess proteolytic activity in tissues associated with chronic inflammation and repeated repair processes. By binding to MMPs in a 1:1 stoichiometric ratio, TIMPs prevent excessive ECM degradation and maintain tissue integrity. Dysregulation of TIMPs, characterized by decreased expression or altered activity and contributing to ECM remodeling and disease progression, is observed in liver pathologies. Conversely, overproduction of TIMPs, especially TIMP-1 and TIMP-2, reduces ECM degradation. TIMPs can influence the behavior of HSCs, as TIMP-1 and TIMP-2 regulate HSC proliferation [51], and TIMP-1 interacts with TGF-β signaling components, potentially enhancing the fibrogenic response [21]. MMPs and TIMPs are considered to play central roles in the development and regression of LF, as evidenced in many studies, and thus represent potential therapeutic targets.

Both MMPs and TIMPs are secreted mainly by HSCs and particularly by aHSCs during liver injury, but other cell types found in the liver also participate, especially immune cells such as KCs. This is related to the fact that different MMPs are involved in the various phases of liver disease initiation, progression and resolution through their multiple actions in fibrosis, inflammation and tissue regeneration, among other processes [47].

Although the development of LF upon chronic exposure to varying toxic stimuli has many common features, different etiologies present specificities regarding the matrisome. For instance, while solid, dense fibrosis is typical in alcoholic liver disease (ALD), NAFLD is characterized by lattice fibrosis, though both conditions involve increased type I and III collagen. ALD tends to show more aggressive centrilobular fibrosis and sclero-hyaline necrosis, while NAFLD frequently begins with pericellular fibrosis in zone 3, often described as having a “chicken-wire” pattern. Moreover, it is well known that ethanol’s first metabolite, acetaldehyde, is highly fibrogenic and enhances the deposition of different ECM components [52,53], while it forms protein adducts by interacting with the ε–amino group of lysine, or with the α amino group of N-terminal amino acids (known to be the case of collagen for many years now) [54]. In recent years, ECM has also been studied as part of the development of MASLD. For instance, integrin-linked kinase (the ECM-integrin signaling axis) has been found to be necessary for the development of diet-induced hepatic insulin resistance in mice [55] and there is in vitro evidence that collagen I increases palmitate-induced lipotoxicity in hepatic cells via integrin-mediated death [56]. Finally, various matrisome components are also specifically relevant to viral hepatitis development. They can act as receptors for viral entry; e.g., during the interaction between syndecan 1 and HCV [57].

In summary, the matrisome is a complex set of proteins that are released by different cell types in the healthy and injured liver. It participates in different processes in liver disease, both in its progression and its resolution, and is considered to be a valuable marker of LF and a therapeutic target for its treatment.

## 4. Collagen: Types, Roles and Biogenesis

Collagen (from the Greek word “kolla”-glue) is the principal component of the ECM and the most abundant protein in mammals, accounting for 12–17% of total protein in mice [58]. The collagen family comprises 28 members (named I–XXVIII, based on their order of discovery), all of which possess a signature triple-helical domain structure. Collagen proteins are divided into two major subtypes: fibrillar and non-fibrillar. These subtypes refer to the fact that they can form different structures, such as long, thin fibers (as in collagen type I), and non-fibrillar structures that are network- or mash-like (as in collagen type IV or VI). Fibrillar collagens, which commonly contain long and uninterrupted tripeptide domain sequences (Gly–XY–)*_n_*, are present in three polypeptide chains (*α*-chains) with approximately 330–Gly–XY–triplets (X is typically proline and Y is typically 4-hydroxyproline). The number of glycine-X-Y repeats correlates with the rigidity of the molecule. In humans, over 42 genes encoding α-chains have been identified for the 28 collagens. The *α*-chains spontaneously assemble into triple-helical homomeric or heteromeric structures, which in turn spontaneously assemble into fibrils. Non-fibrillar collagens differ from fibrillar collagens in how their triple-helical regions are organized: rather than long uninterrupted sequences, they contain short stretches of the repeating (Gly–XY–)*_n_* motifs or longer repeats of (Gly–XY–)*_n_* that are interrupted by non-collagenous sequences. As a result, these proteins do not assemble into highly ordered fibrils.

Collagen imparts tensile strength and elasticity to the ECM, enabling it to withstand mechanical forces; however, different types of collagens have specific roles in various tissues and organs. For example, collagen type I, the most prevalent form of collagen (as seen in mice [58]), is a major component of the ECM of connective tissues, such as skin, tendons, and ligaments, where it is organized into complex, regular patterns that give these tissues their characteristic mechanical properties, ultimately accounting for 70–90% of total body collagen. Collagen type II is the main structural component of cartilage, while type III is found in the skin, blood vessels, and internal organs. Collagen type IV is a major component of the basement membrane, a thin layer of connective tissue that separates epithelial cells from underlying tissues.

In addition to its structural role, collagen also plays a key role in the signaling and regulation of ECM functions, as it can interact with various molecules, such as growth factors, enzymes, and other ECM components, to modulate their activity and regulate their effects on surrounding cells [59].

As explained using collagen type I as an example, the biosynthesis of collagens starts with the transcription of the genes encoding for pro-α1 and pro-α2 chains, *COL1A1* and *COL1A2*. Multiple transcription factors have been implicated in the control of collagen gene transcription, both under basal conditions and in the setting of liver injury, with additional regulators continuing to be identified, including ZNF469, which modulates collagen production in LF associated with MASLD [60]. Once generated, COL transcripts move into the cytoplasm, interact with ribosomes, and are translated to a long uninterrupted collagenous peptide product known as pre-procollagen chain [61]. La ribonucleoprotein domain family member 6 (LARP6), an RNA-binding protein, is specifically involved in post-transcriptional regulation, especially in the stabilization of mRNAencoding collagen type I for the translational process [62]. On the other hand, miRNAs, and particularly miRNA29, inhibit translation by forming a complex with the mRNA [63,64].

**Figure 2:**
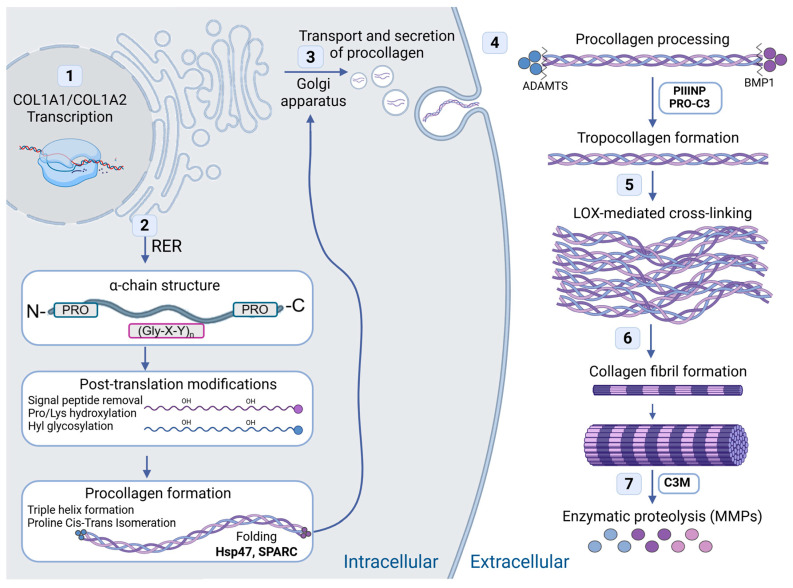
The process of collagen synthesis, secretion, and turnover, illustrated with the example of collagen 1. Representation of the mechanisms involved in collagen expression (1), folding and maturation (2,3), cleavage (4), assembly (5,6) and degradation (7). The biomarkers based on collagen are also shown (PIIINP and PRO-C3). Created in BioRender. Benavides, C. (2026) https://BioRender.com/tmqix3k.

Following peptide formation, the pre-procollagen undergoes post-translational modifications in the rough ER (Figure 2). First, its terminal part (the signal peptide) is cleaved. Second, with the support of ascorbic acid and multiple cofactors, proline and lysine residues are hydroxylated via collagen prolyl-hydroxylases and lysyl-hydroxylases, respectively [65]. A hydroxyl group is added at the 4th-carbon or the 3rd-carbon of proline residues via collagen prolyl 4-hydroxylases or prolyl 3-hydroxylases to yield 4-hydroxyproline or 3-hydroxyproline, respectively. In addition, various carbons of the aliphatic side chain of lysine residues are hydroxylated through the action of collagen lysyl 5-hydroxylases. Third, selected hydroxylysyl residues are glycosylated by galactosyltransferases with the disaccharide glucose-galactose [66], giving rise to a molecule known as procollagen.

The following step is the formation of the pro-collagen triple helix. In the case of collagen type I, three chains of procollagen, two of pro-α1 and one of pro-α2, are folded in a zipper-like fashion, while other types of collagens, such as type II and III, assemble as homotrimers with three identical α chains [61]. This process is facilitated by multiple folding catalysts within the rough ER, such as glucose-regulated protein 78 (GRP78; also known as immunoglobulin binding protein, BiP; or heat shock protein family A (HSP70) member 5, HSPA5), GRP94, and protein disulfide isomerase (PDI) [67]. Peptidyl-proline isomerases (PPIases), including FK506-binding protein (FKBP) 10, FKBP11, and FKBP14, are rate-limiting enzymes, as the number of proline residues in the collagen molecule is relatively high [68]. Furthermore, heat shock protein 47 (HSP47) is a collagen-specific chaperone that is pivotal to the maturation of the helix [69]. Finally, the last step occurs in the Golgi apparatus, where procollagen molecules are assembled into secretory vesicles for secretion into the extracellular space.

Before the fibrillar collagen molecule is incorporated correctly into fibrils, it must be properly processed. This process of maturation is achieved extracellularly and includes cleavage of peptides (propeptides) from procollagen by the specific N-terminal and C-terminal propeptidases to yield tropocollagen [70]. The enzymes involved are members of the ADAMTS family [71] or BMP-1 [72], depending on whether the N- or C-terminal end of the molecule is being cleaved and on the specific collagen.

Tropocollagen then undergoes a process of maturation and self-assembles into fibrils through the activity of the lysyl oxidase family, which includes lysyl oxidase (LOX) and lysyl oxidase-like (LOXL) 1 to 4 [73]. These copper-dependent enzymes possess a conserved C-terminal catalytic domain and oxidize primary amines to reactive aldehydes. Their activity is regulated by hypoxia, oxidative stress, and inflammation. Specifically, they selectively oxidize collagen ε-amino groups of lysine and hydroxylysine residues to allysine aldehydes (LysAld), which initiate intra- and intermolecular covalent cross-linking of ECM proteins, including elastin and collagen, thereby conferring tensile strength and structural stability to the ECM. Intracellular actions of some of the LOX members (such as LOXL2) have also been described [74]. The LOX family can be classified into two subfamilies based on their N-terminal structure: Subfamily 1 (LOX and LOXL1) and subfamily 2 (LOXL2, LOXL3 and LOXL4) [74]. Interestingly, LOX and LOXL1 are proteolytically processed (and thereby activated) by BMP-1/Tolloid-like metalloproteinases, proteolytic enzymes whose wide repertoire of substrates are mainly ECM and ECM-related proteins.

The dysregulation of members of the LOX family has been linked to a number of health issues, such as fibrosis in various tissues (including the lung and liver), and the development of aneurysms and arterial hypertension [75]. In addition, LOX is upregulated in several types of cancer and has been linked to increased invasion and metastasis [76], and may also contribute to neurodegeneration [77]. Given this evidence, drugs are being developed to target LOX for the treatment of various types of cancer and fibrosis, and LOX and LOX-mediated cross-links are potential biomarkers for a variety of diseases [78,79]. In the context of CLD, these enzymes are closely related to LF—HSCs and portal fibroblasts are their major cellular sources in the normal liver and shortly after injury [80]. LOX and related enzymes in HSCs directly affect other cell types in the liver; in this sense, it has recently been shown that targeting LOXL1-expressing HSCs inhibits fibrogenesis and sinusoid angiogenesis via the LOXL1/RUNX1/VEGFA axis during the progression of LF [81].

Transglutaminases (TGs), whose activity depends on Ca^2+^ and catalyze the linking of lysine to glutamine residues, also participate in collagen cross-linking. TG2 is the most ubiquitously expressed enzyme of this family, including the liver, but is largely retained in an inactive state intracellularly. Its secretion is carefully regulated—in particular, by myofibroblasts and endothelial cells—following cellular damage, where it is activated by high extracellular Ca^2+^ concentrations [82].

Finally, collagen is also subject to degradation, a process involving extracellular and intracellular pathways. The former are mediated by membrane-bound and secreted proteolytic enzymes such as MMPs, while, in the latter, relatively intact collagen fibrils are internalized through phagocytosis, macropinocytosis or uPARAP/Endo180-mediated endocytosis and subsequently degraded by lysosomal cysteine proteases [83].

## 5. Collagen in Liver Fibrosis: Same but Different

The collagen found in a normal healthy liver is mostly fibrillar types I, III and V, is localized mainly in the interstitial matrix, within the space of Disse, at the portal tract and the central vein, and is produced by (myo-)fibroblasts, primarily serving to support tissue structure [84]. The basement membrane type IV collagen is also present, produced principally by endothelial cells and qHSCs localized in the sinusoidal walls and in the vessel walls adjacent to the bile duct epithelial cells. Its main role is to support specialized polarized cells in order to allow the diffusion of molecules between blood and liver endothelia and epithelia. As LF develops and progresses, the number and distribution of collagens change [84,85]. The ECM deposited in LF is interstitial and mainly consists of fibril-forming type I and type III and minor quantities of type V collagen. In cirrhotic livers, increments of up to 10-fold—corresponding to liver mass of collagen types I, III, and V—have been registered, while increases in collagen type IV and other non-collagenous ECM components, such as fibronectin, have been found to be up to 6-fold [85]. In CCl_4_-induced rat liver fibrosis model, quantitative analysis of Sirius red staining revealed that total collagen deposition in the liver increased as fibrosis progressed: 4.94% of the total tissue in liver sections at 4 weeks, 8.25% at 6 weeks, and 9.11% at 8 weeks [86]. It is unclear which are the most important proteins in the ECM, and, hence, those that should be specifically addressed in fibrosis. Collagen types I and III are the most abundant, followed by types IV, V, and VI, nidogen, laminin, fibronectin, biglycan, mimican, versican, decorin, lumican, and elastin, to name but a few, all of which are clearly altered in quantity and quality during fibrosis. The initial histological changes and developmental patterns of LF (major fibrogenic cell type, major ECM components, fibrosis pattern) vary among different CLDs, such as biliary fibrosis, viral hepatitis, metabolic and ALD.

In addition to the increase in the amount of collagen deposited during liver fibrogenesis, the alignment and cross-linking of collagen fibrils also contribute significantly to ECM stiffness. LOX and LOX-like enzymes, which are mainly synthetized by aHSCs, are the predominant cross-linking enzymes, as the participation of TG2 does not seem to be relevant [87]. LOX has also been shown to interact with fibronectin, which in turn increases the catalytic activity of LOX, thereby increasing the cross-linking of collagens, and subsequently, matrix stiffness [88]. LOXL1 is constitutively expressed in a spatiotemporal expression pattern that is highly upregulated in the foetal state but is limited in healthy adult livers [89]. However, during the late stages of fibrogenesis, LOXL1 is upregulated in a parallel pattern, with an increase in collagen type I. Inhibition of LOXL1 expression arrests LF progression in cirrhosis by reducing elastin cross-linking [90]. LOX helps to stabilize collagen in LF; the proportion of insoluble collagens was shown to increase from 5.7% in healthy tissue to 14.7% and 19.1% in C57Bl/6J mice treated with CCl_4_ for 3 and 6 weeks, respectively, while soluble collagens decreased from 92% in healthy controls to 84% and 79% in fibrotic tissue at 3 and 6 weeks [91]. Treatment with the LOX inhibitor β-aminopropionitrile (BAPN) in the same animal model decreased collagen stability and facilitated LF reversal, as it prevented the increase in highly cross-linked (insoluble) collagen in the advanced stages (from week 3 to 6) of LF progression [91]. Besides the aforementioned animal models, elevated expression and activity of LOX family members are also observed in sera of patients with LF and cirrhosis [92], and multiple studies show similar upregulation in fibrotic livers of patients with Wilson’s disease, primary biliary cholangitis, HCV infection, and NASH (more recently renamed metabolic dysfunction-associated, or MASH) [89]. Due to the abundant substrate concentration and effective catalytic activity of LOX—in combination with the slow rate of subsequent condensation reactions into crosslinks between proteins—there is an accumulation of extracellular allysine during fibrogenesis. Therefore, extracellular allysine of oxidized collagen may represent a particular target for fibrogenesis [93,94]. Specifically, LOXL2 targeting has emerged as an attractive antifibrotic strategy; however, there is a lack of clinical evidence to confirm its usefulness. LOXL1, another member of the LOX family, is thought to promote the cross-linking of elastic proteins [95]. Suppressing the expression of LOXL1 through silencing techniques can prevent the progression of cirrhosis by reducing the cross-linking of elastin in the hepatic ECM [90]. Other collagen-related mediators have also been linked to LF. DDR2 mediates the expression of the type IV collagenase (gelatinase) MMP-2 in aHSCs in response to type I collagen during liver injury [96], as well as the expression of membrane type-1 MMP (MT1-MMP, MMP-14) in fibroblasts when stimulated by a collagen-rich environment [97]. Similarly, extracellular TG2-dependent matrix cross-linking has been closely linked with the pathogenesis of LF [98,99].

During ECM turnover, smaller or larger fragments of the ECM are released into the circulation, after which they constitute biomarkers of fibrogenesis or fibrolysis (fibrosis resolution), depending on the characteristics of the fragments, as some are formation-related peptides, while others are degradation-related. Such serum biomarkers have a distinct advantage over indirect biomarkers and scores, such as ALT, AST and FIB-4, in that they more accurately reflect the dynamics of the fibrotic processes. Collagens, including propeptides of collagens, and other components of ECM have been proposed as non-invasive biomarkers of LF [84]. While collagen type I is highly abundant in hard tissues, such as bones, the expression of type III collagen is a characteristic of soft tissues and correlates with the content of (myo)fibroblasts. Consequently, type III collagen is a more accurate indicator of fibrotic processes than type I, which makes the type III (pro) collagen peptides promising prognostic biomarkers for LF. At present, the most validated and accurate biomarkers for the measurement of type III collagen formation are the N-terminal propeptides PIIINP and PRO-C3 biomarkers [100,101]. Both PRO-C3 and PIIINP quantify the formation of type III collagen by targeting the N-terminal propeptides. The PIIINP antibody targets an internal unknown epitope within the N-terminal propeptides, whereas PRO-C3 measures true formation by specifically targeting the site at which ADAMTS2 cleaves off the propeptides. Blood levels of PIIINP, together with hyaluronic acid and TIMP-1, are used for the so-called “enhanced liver fibrosis (ELF™) test”, a score which estimates the extent of LF [102]. Clinical studies indicate that this test is sensitive, specific and reproducible, suggesting that it can be used either alongside or independently of liver biopsy to assess a range of CLDs. The superiority of ECM-based biomarkers over the traditional ones has been reported many times. In one study, baseline ALT and AST did not predict changes in fibrosis stage, whereas elevated baseline levels of PRO-C3 were predictive of fibrosis progression in patients with HCV [101]. Additionally, within a phase II trial investigating aldafermin (an analog of fibroblast growth factor 19) as a potential treatment for MASH, both PRO-C3 and ELF™ were able to identify histological responders, whereas ALT could not [103]. Indeed, direct biomarkers of LF, such as ELF™ and PRO-C3, are diagnostically superior to indirect indices, such as APRI and FIB-4, when employed to detect advanced LF [104,105]. PRO-C3 and ELF™ score have been proposed as useful markers for fibrosis staging and prognosis and can be used to discriminate advanced fibrosis and cirrhosis in patients with primary sclerosing cholangitis [106]. The broad utility of ECM-based biomarkers presents some challenges, as their circulating levels can be influenced by some of the common comorbidities in these patients, and other tissues can be predominant sources under certain conditions. For example, the ability of ELF™ to represent fibrogenesis—largely attributable to PIIINP—can be partly obscured by the contribution of TIMP-1, as 75% of that which is present in serum is released from blood cells such as platelets [107].

## 6. The Role of Matrisome in Signaling: When Mechanics Meets Biochemistry

Mechanosignaling is fundamental for liver homeostasis. Increased cytoskeletal tension and ECM rigidity are sensed through integrins (a diverse family of cell surface receptors) and mechanosensitive ion channels (such as Piezo1 and TRPV). These signals converge on key pathways that regulate different aspects of liver homeostasis (such as hepatic regeneration, fibrogenesis, immune-cell recruitment and angiogenic remodeling) by acting on both parenchymal and non-parenchymal cells.

**Figure 3:**
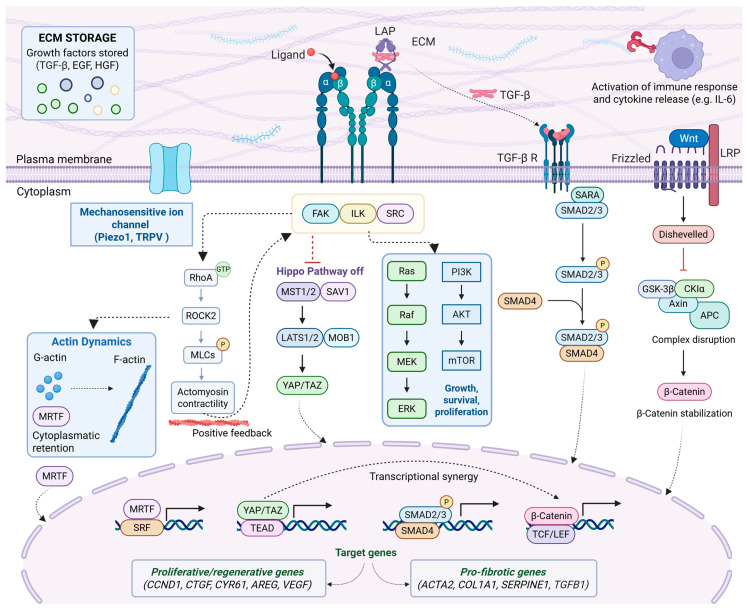
Signaling by integrins participates in the activation of HSCs. Rho/ROCK/MRTF/SRF, Hippo/YAP/TAZ, MAPK cascades, TGF-β mediated SMAD and Wnt/β catenin transcription factor pathways are shown. ACTA2, smooth muscle actin; AREG, amphiregulin; COL1A1, collagen type I alpha 1; CTGF, connective tissue growth factor; ECM, extracellular matrix; EGF, epidermal growth factor; FAK, focal adhesion kinase; HGF, hepatocyte growth factor; ILK, integrin-linked kinase; LAP, latency-associated peptide; LRP, low-density lipoprotein receptor-related protein; MLC, myosin light chain; MOB1, Mps one binder; MRTF, myocardin-related transcription factor; SARA, anchor for receptor activation; SAV1, protein salvador homolog 1; SMAD, mothers against decapentaplegic homolog; SRF, serum response factor; TCF/LEF, T cell factor/lymphoid enhancer factor; TEAD, transcription enhancer with TEA domain; TGF-β, transforming growth factor beta; VEGF, vascular endothelial growth factor. Created in BioRender. Martínez arroyo, O. (2026) https://BioRender.com/iie86i0.

Integrins, the primary mediators of cell–ECM adhesion, exist as heterodimers formed through non-covalent interactions between 18 α- and 8 β-subunits, generating 24 distinct αβ combinations. On the basis of their main ligands, integrins are classified into RGD (arginine–glycine–aspartic acid)-binding integrins (α3β1, αvβ1, αvβ3, αvβ5, αvβ6, αvβ8, and αIIβ3) that interact with proteins containing the RGD peptide, such as fibronectin, vitronectin, fibrinogen, and thrombospondin-1, collagen-binding integrins (α1β1, α2β1, α10β1, and α11β1) that bind to both fibrillar and nonfibrillar collagens, and laminin-binding integrins (α3β1, α6β1, α7β1, and α6β4). Signaling through integrins tightly regulates LF, as recently reviewed by Sharip A et al. [108]. In HSCs, integrins α8β1 and α11β1 are selectively expressed and contribute to fibrogenic activation. More broadly, integrins in the liver regulate multiple cellular processes—including adhesion, migration, survival, and fibrogenesis—through the activation of diverse signaling pathways in a cell type–dependent manner. Clustered in focal adhesions, they recruit integrin-linked kinase (ILK), focal adhesion kinase (FAK) and proto-oncogene tyrosine-protein kinase Src, thereby activating the classic RAS-RAF-MEK-ERK and PI3K-AKT-mTOR cascades to promote anabolic growth, survival and proliferation [109]. Concurrently, integrin engagement stimulates RhoA–ROCK–mediated actomyosin contractility, amplifying FAK signaling in a positive feedback loop that refines focal adhesion maturation and strength. Mechanotransduction also involves Hippo-YAP/TAZ and Wnt/β-catenin cascades [110]. When MST1/2-LATS1/2 kinase activity is suppressed, YAP and TAZ are able to translocate into the nucleus; there, they partner with TEA domain transcription factor family (TEAD) to enhance the expression of pro-proliferative genes—e.g., cyclin D1 (*CCND1)*, connective tissue growth factor (*CTGF*), cysteine-rich angiogenic inducer 61 (CYR61), and amphiregulin (*AREG*)—which drives tissue restructuring and regeneration, and pro-fibrotic genes (such as *ACTA2* and *COL1A1*) depending on the cell type. Regarding canonical Wnt/β-catenin signaling, disruption of the complex Axin/GSK3β stabilizes β-catenin, which then cooperates with T-cell factor (TCF) and is further potentiated by YAP/TAZ activation, thus providing transcriptional synergy [109]. Signaling through integrins in HSCs is schematically shown in Figure 3.

ECM-DAMPs (damage-associated molecular patterns) are also worthy of mention [111]. Newly synthetized or remodeled ECM components, such as fragmented hyaluronan or collagen, act as DAMPs that bind to pattern recognition receptors such as Toll-like receptors (TLRs) on immune cells including macrophages, monocytes, dendritic cells and T cells, as well as with endothelial cells. This drives chronic inflammation and activates immune cells, contributing to liver pathogenesis.

Non-core matrisome components such as ECM-affiliated proteins galectin-3 (GAL3), ANXA2 and SPARC also function as molecular modulators of cell–matrix adhesion. Finally, the ECM also regulates different cellular processes due to its capacity to store many growth factors (like HGF, EGF, TGF-β) for controlled release (Figure 3). This is crucial for promoting tissue repair and promoting (or limiting) fibrosis.

## 7. Keeping the Matrisome in Line as a Promising Antifibrotic Strategy

The therapies proposed specifically for LF have focused largely on reducing fibrogenesis by targeting cells associated with excessive ECM formation, while there is much less understanding of how fibrolysis becomes insufficient to match the overproduction of ECM during LF or how the immune system can be stimulated to activate cellular mechanisms that degrade the excessive ECM. Actually, macrophages are emerging as important upstream regulators of LF and direct effectors of fibrosis resolution, controlled by specific subtypes, such as M1 and variant M2 macrophage subsets. ECM stiffness, besides ECM excess deposition, also needs to be considered in relation to mechanotransduction as a potential antifibrotic target. While traditionally viewed as an endpoint, matrix stiffening is now known to develop early during fibrosis initiation, and to make a strong contribution to disease progression through mechano-activation of myofibroblasts (and other cell types).

It is also important that antifibrotic strategies focus on interrupting the pro-fibrotic cross-talk that occurs between liver myofibroblasts (HSCs, for that matter) and other parenchymal and non-parenchymal cell types.

Directly targeting collagen homeostasis in LF has been explored in numerous preclinical studies, but its clinical translation is still pending. Collagen metabolism is complex, and is composed of several sub-processes. Transcriptional/translational inhibitors can be used to halt collagen synthesis, as shown in studies in rodents with LF; the deposition of collagens was shown to be reduced by delivery to the liver of the small interference RNA (siRNA)-targeting COL1A1 in lipid-like nanoparticles, or by knockdown of collagen type I expression using a short hairpin RNA (shRNA), which ameliorated LF [112,113]. The formation of collagen peptides can be attenuated by inhibiting mRNA-binding protein LARP6, as seen in cultured fibroblasts, ex vivo precision-cut liver slices, and in vivo models of LF [62,114]. Further in the process of collagen synthesis, post-translational modification of collagens at the rough ER is a promising therapeutic target, with prolyl 4-hydroxylases showing particular potential. These enzymes are members of the Fe(II)- and α-ketoglutarate-dependent dioxygenase family and are responsible for attaching hydroxyl groups at proline residues of collagens. Metal chelators, mimetics of α-ketoglutarate or collagen peptides can be used for the inhibition of prolyl 4-hydroxylases. In this sense, HOE-077 (aka lufironil), a prodrug of pyridine-2,4-dicarboxylic acid and an analogue of α-ketoglutarate, was shown to inhibit collagen synthesis in rat livers [115]. Collagen helix-formation inhibitors have also been explored. For example, BMS-986263, a retinoid-conjugated lipid nanoparticle siRNA designed to silence HSP47, was assessed in patients with advanced LF secondary to HCV infection. This 36-week placebo-controlled phase 2 clinical trial revealed improved liver pathology scores [116]. Another phase 2 clinical trial (NCT04267393) is ongoing and aims to evaluate the effectiveness of BMS-986263 in adults with compensated cirrhosis due to NASH [117].

The inhibition of collagen fibril/fibrin-formation has also been assessed in multiple preclinical studies in which different members of the LOX family and TGs have been targeted. Among the different strategies employed, small-molecule inhibitors of members of the LOX family have displayed antifibrotic capacity [91,118,119]. Specific antibodies against LOXL2 have shown efficacy in vivo; e.g., the inhibitory monoclonal antibody AB0023 exerted beneficial effects during early treatment in a mouse model of mild LF [120], though it is yet to be tested in clinical trials. In contrast, simtuzumab (formerly GS-6624), another anti-LOXL2 antibody (Gilead Sciences), failed to improve LF in several phase II clinical trials, namely in compensated cirrhotic NASH patients [121], patients with primary sclerosing cholangitis, a chronic cholestatic disease [122], and advanced LF patients infected with HCV, HIV, or HCV-HIV [123], as there was no significant improvement after 96-week treatment versus placebo (Table 1). Collagen receptors, particularly DDRs, have also been the subject of research, but their beneficial effects are questionable. While targeting DDR2 with shRNA alleviated collagen deposition in rats with ALD [124], DDR2-knockout mice actually displayed more LF after CCl_4_-induced chronic liver injury, suggesting a role for DDR2 in attenuating fibrogenesis [125]. Alternatively, DDRs are inhibited by multikinase inhibitors, such as imatinib, which can simultaneously block multiple tyrosine kinases, including both DDR1 and DDR2. A clinical study to evaluate the efficacy of imatinib in advanced LF patients has been registered (NCT05224128) [126].

Finally, strategies based on ECM degradation achieved through two possible approaches have also been investigated: namely, an increase in the expression/activity of MMPs or the inhibition of TIMPs. In this context, the therapeutic potential of stimulated expression of MMP-1, MMP-8 or MMP-9 using viral or plasmid vectors has been widely illustrated in various animal models [127,128,129]; however, the implementation of this approach in patients is still a challenge and currently unfeasible. TIMP-1 and TIMP-2 inhibition through siRNA- or shRNA- targeting, or the use of antisense oligonucleotides, promotes ECM degradation and has an antifibrotic action in rodent models [130,131,132].

Beyond MMP-mediated ECM degradation, intracellular pathways involved in ECM turnover merit exploration as part of the search for antifibrotic targets [133], though it has been assessed in few preclinical studies to date. One recent study suggested that pericentral senescent hepatocytes secrete the glucoprotein clusterin, which induces the proliferation and migration of mesothelial cells that endocytose fibers through the receptor LRP2 (low-density lipoprotein receptor-related protein 2), thus reducing LF [134]. In addition, LSECs are a promising cell type warranting further study, as they are professional scavenger endothelial cells capable of clearing many types of circulating macromolecules in plasma, including components of the ECM, such as TGF-β -induced, 68 kDa (TGFBi) and periostin (POSTN) through scavenger receptors stabilin-1 and stabilin-2 [135]. Nevertheless, there has yet been no clinical translation of these antifibrotic approaches.

Non-collagen components of the matrisome have also been the subject of intense preclinical research. For instance, exogenous decorin, a small leucine-rich secreted proteoglycan, accelerates liver regeneration after partial hepatectomy in fibrotic mice [136]. However, only a few molecules have reached the clinical stage of research. In this regard, integrins are an attractive antifibrotic target that has been assessed in clinical settings. PLN-74809, developed by Pliant Therapeutics, is an oral small-molecule dual inhibitor of αvβ1 and αvβ6 integrins whose aim is to prevent the activation of latent TGF-β1 and TGF-β3, thus reducing LF. In fact, it has shown some promising results in patients with primary sclerosing cholangitis (Table 1). Bexotegrast has been granted fast track designation and orphan drug designation by the FDA for IPF. Additionally, it has received Orphan Drug Designation for primary sclerosing cholangitis from both the FDA and EMA [108]. The same company has developed PLN-1474, a small selective inhibitor of the αVβ1 integrin and reported testing in Phase I clinical trials. In addition, galectin-3 inhibitors have been tested in humans. One clinical study assessed GR-MD-02, a polysaccharide-based compound administered intravenously in three different dosing regimens and compared it to placebo in patients with NASH with advanced LF [137]. Notably, only in the cohort with the highest dose (8 mg/kg) a significant reduction in FibroTest scores was observed. This finding was examined further in patients who received GR-MD-02 8 mg/kg biweekly for 16 weeks. No differences were observed between treatment and placebo in iron-corrected T1 (cT1) mapping (non-invasive MRI-based diagnostic biomarker used to measure hepatic fibro-inflammatory activity) and liver stiffness evaluated by magnetic resonance elastography or shear-wave elastography [138].

In addition, while fibroproliferative diseases differ in their properties and underlying pathogenic mechanisms, knowledge derived from preclinical and clinical studies evaluating antifibrotic (and for that matter matrisome-based) approaches in other such disorders, including idiopathic pulmonary fibrosis and scleroderma, may still be valuable.

Finally, changes in the matrisome in the injured liver are increasingly recognized as a driver, rather than just a byproduct, of disease progression. For example, specific matrisome gene signatures, such as microfibrillar associated protein 4 (MFAP4)-associated profiles, define subgroups of patients with LF and reflect active pathogenic processes rather than passive scarring [38]. Moreover, the quantitative and qualitative changes in the matrisome during LF vary depending on the stage and etiology of the disease [139]. In light of all this, it is vital to consider the active role of the matrisome during the search for therapeutic targets and diagnostic tools for LF.

## 8. Conclusions and Future Perspectives

Given its global burden, CLD and its end-stages, cirrhosis and liver cancer, represent a critical area of clinical interest. A key prognostic histological feature in CLD is the presence and severity of LF, but effective pharmacotherapy to treat it is a major unmet medical need. The matrisome is a collective term for the ensemble of ECM proteins and a myriad of associated factors. The involvement of matrisome remodeling in LF highlights an important direction for future research. A comprehensive understanding of ECM and ECM-cell interactions could boost the development of novel diagnostic and therapeutic strategies, ultimately improving outcomes for individuals affected by CLD. In this regard, antifibrotic therapies for LF that target the hepatic matrisome should focus on ECM composition, cross-linking, and mechanotransduction. The antifibrotic efficacy of drugs that interfere with the matrisome at different levels has been demonstrated in preclinical studies, but translation to clinical trials is still limited, as only simtuzumab (LOXL2 inhibitor antibody), imatinib (small-molecule inhibitor against DDRs), bexotegrast (integrin inhibitor), belapectin (galectin 3 inhibitor) and BMS-986263 (siRNA-targeting HSP47) have been or are being evaluated in clinical trials of various CLDs.

Several reasons can explain the failure of clinical trials and preclinical studies concerning the ECM in LF. Firstly, cross-linking mechanisms are redundant; when LOX is blocked, other enzymes, such as TGs, and advanced glycation end products (AGEs) continue to contribute to the stiffening of the ECM, thus sustaining LF, which may explain why LOX-targeting therapies have failed in clinical trials. In a similar vein, inhibiting one collagen subtype may increase others. Finally, many trials enroll patients with advanced LF or cirrhosis, at which stage collagen is already heavily crosslinked and biologically stable. In these circumstances, simply reducing new collagen synthesis may not be sufficient to reverse the existing LF.

With respect to a technological approach, besides conventional inhibition using small-molecule inhibitors or antibodies, gene therapy is currently the most promising strategy to knockdown the expression of collagens and collagen-associated molecules. Therapeutics for LF face many challenges; for example, a specific delivery method is necessary to transport the therapeutic agents directly to the targeted locations within the liver and limit their off-target adverse reactions. This is highly relevant given that collagens in other tissues, such as skin, bone, and cartilage, are fundamental. Furthermore, pharmacological interference with the matrisome in LF may have several drawbacks, including inflammatory amplification, immune dysregulation, destabilization of the physiological liver architecture and impaired tissue regeneration and vascular injury.

## Figures and Tables

**Table 1:** Clinical trials that assess the liver antifibrotic efficacy of drugs whose targets are related to the hepatic matrisome. Reference from ClinicalTrials.gov. LF, liver fibrosis; IV, intravenous; NASH, non-alcoholic steatohepatitis; PSC, primary sclerosing cholangitis.

Simtuzumab	
Drug mechanism	Humanized monoclonal antibody that inhibits LOXL2.
Reference	NCT01672866
Disease	NASH-associated advanced LF (no cirrhosis).
Design	Phase 2b, randomized, double-blind, placebo-controlled trial in adults with advanced LF due to NASH, but without cirrhosis.
Objective	To evaluate if it prevents histologic progression of LF and cirrhosis.
Status/results	Terminated; no efficacy advantage seen.
Reference	NCT01672879
Disease	NASH-associated compensated cirrhosis.
Design	Phase 2b, randomized, double-blind, placebo-controlled study in adults with compensated cirrhosis due to NASH.
Objective	To evaluate safety and efficacy in cirrhotic NASH patients.
Status/results	Terminated/results posted; no benefit in cirrhosis endpoints.
Reference	NCT01672853
Disease	PSC with LF.
Design	Phase 2, placebo-controlled study in patients with PSC.
Objective	To evaluate efficacy in slowing LF progression.
Status/results	Terminated. No significant clinical benefit in LF or progression to cirrhosis.
Imatinib	
Drug mechanism	Tyrosine kinase inhibitor.
Reference	NCT05224128
Disease	Advanced LF.
Design	Phase 1/Phase 2, randomized, double-blind, placebo-controlled trial comparing standard of care plus imatinib vs. standard of care plus placebo (parallel-group interventional model).
Objective	To evaluate safety and efficacy in moderate/severe LF (grade F3–F4).
Status/results	Status: “Unknown”. No published efficacy results available.
BMS-986263	
Drug mechanism	Retinoid-conjugated lipid nanoparticle that delivers siRNA designed to inhibit synthesis of HSP47 protein.
Reference	NCT03420768
Disease	Advanced LF (METAVIR F3–F4) in adults who achieved sustained virologic response after hepatitis C infection.
Design	Phase 2, randomized, double-blind, placebo-controlled study. Parallel groups receiving once-weekly BMS-986263 45 mg, BMS-986263 90 mg, or placebo, administered IV for 12 weeks.
Objective	To evaluate efficacy, safety, pharmacokinetics and pharmacodynamics.
Status/results	Completed. Modest improvements in LF: at week 12, ≥1 METAVIR stage improvement in ~21% of patients on 90 mg, ~17% on 45 mg, and ~13% on placebo, with some Ishak score improvements at 90 mg.
Reference	NCT04267393
Disease	Compensated cirrhosis due to NASH.
Design	Phase 2, randomized, double-blind, placebo-controlled, parallel-group study vs. placebo (typically with similar weekly IV dosing regimens).
Objective	To assess safety and effectiveness in reversing LF and improving liver histological features in NASH-related cirrhosis.
Status/results	Terminated. Lack of short-term efficacy, and no significant improvement in relevant endpoints such as hepatic venous pressure gradient or LF measures compared with placebo.
PLN-74809 (Bexotegrast)	
Drug mechanism	Inhibits αvβ6 and αvβ6 integrins.
Reference	NCT04480840
Disease	PSC and suspected moderate-to-severe LF.
Design	Phase 2a, multicenter, randomized, double-blind, dose-ranging, placebo-controlled study.
Objective	To evaluate safety, tolerability, pharmacokinetics and exploratory efficacy.
Status/results	Terminated. Reduced ELF scores and PRO-C3 levels at week 12 vs. placebo, with significant differences at 160 mg. Dose-dependent reduction in alkaline phosphatase (ALP) blood levels.
GR-MD-02 (Belapectin)	
Drug mechanism	Galectin 3 inhibitor
Reference	NCT01899859
Disease	Biopsy-proven NASH with advanced fibrosis.
Design	Phase 1, partially blinded, multicenter, randomized, dose-ranging, placebo-controlled study (administration as a single IV infusion followed by 3 additional weekly infusions starting 28 days after the first dose).
Objective	To evaluate safety, tolerability, pharmacokinetics and exploratory efficacy.
Status/results	Terminated. Fibrotest Score significantly reduced in patients on the highest dose of the drug tested (8 mg/kg) vs. placebo.
Reference	NCT02421094
Disease	Biopsy-proven NASH with advanced fibrosis.
Design	Phase 2, single center, blinded, randomized, placebo-controlled study (administration for 16 weeks).
Objective	To evaluate efficacy in liver inflammation, fibrosis and stiffness.
Status/results	Terminated. No differences in iron-corrected T1 (cT1) or liver stiffness.

## Data Availability

No new data were created or analyzed in this study.

## Abbreviations

The following abbreviations are used in this manuscript:

ALD	Alcoholic liver disease
CLD	Chronic liver disease
ECM	Extracellular matrix
HSC	Hepatic stellate cell
KC	Kupffer cell
LF	Liver fibrosis
LOX	Lysyl oxidase
LSEC	Liver sinusoidal endothelial cell
MASLD	Metabolic dysfunction–associated steatotic liver disease
MMP	Metalloproteinase
NAFLD	Non-alcoholic fatty liver disease
NASH	Non-alcoholic steatohepatitis
TGF-β	Transforming growth factor beta
TIMP	Tissue inhibitor of metalloproteinases
TG	Transglutaminase
